# DairyCoPilot—Automated data compilation and analysis tools for DairyComp data assets

**DOI:** 10.1371/journal.pone.0297827

**Published:** 2024-04-18

**Authors:** Srikanth Aravamuthan, Dorte Dopfer, Emil Walleser

**Affiliations:** Department of Medical Sciences, School of Veterinary Medicine, University of Wisconsin, Madison, WI, United States of America; Michigan State University, UNITED STATES

## Abstract

Modern dairy farm management requires meaningful data and careful analysis to maximize profitability, cow health, and welfare. Current data platforms, such as DairyComp, lack robust integrated data analysis tools. Producers and consultants need dedicated tools to turn collected data sets into assets for informed decision-making processes. The DairyCoPilot app allows users to rapidly extract health and production data from DairyComp, then compile and analyze the data using a menu-driven point-and-click approach. Prospects for training consultants in applied data analysis skills make DairyCoPilot a tool to identify farm management bottlenecks with less time spent for data analysis, improving cow health, and dairy production. The DairyCoPilot Dashboard R Shiny application is published using RStudio Connect: https://connect.doit.wisc.edu/dairy-copilot/.

## Introduction

Managing dairy cows has become a data-intensive practice [1, 2]. Increased number of cows per farm combined with enhanced data recording has expanded the availability of data for producers and consultants to make informed decisions [3]. Deriving value from farm data and cow records requires software and trained users resulting in so-called ‘data assets’ aligned with evidence-based decision-making. Examples of dairy record management software are DairyComp, BoviSync, and animal by MILC. The current project will focus on data compilation and analysis from DairyComp (DC), a management package that is utilized by about 60% of dairy herds in the US [4].

According to its promoters, DC has become one of the most popular dairy herd management software tools [5]. The software allows producers to store information about individual cows and cow-level events in a “cow card” to examine the cows’ entire history, their health, reproduction, and production records. Cow management worksheets, monitoring reports, reproduction management, and other tools are integrated directly into DC. A set of built-in analysis tools for graphic displays of trends in health events, reproduction, and milk production assist with cow monitoring. However, DC does not currently possess internal tools for statistical inference controlled for confounders or provide a simple way to extract cow information for data analysis making it difficult for dairies to gain insights. Detailed health data analysis provides opportunities for adding value using knowledge about production and health challenges. Previously, detailed health analyses using DC data required significant manual data processing both in DC and in spreadsheet tool of user choice.

The proposed DairyCoPilot app is time efficient for single and repeated applications. We provide a singular, automated workflow for extracting health and event information from DC to DairyCoPilot followed by a graphical user interface application deployed on an R Shiny server. The initial setup requires minimal time in DC. The R Shiny application uploads the extracted file from DC to download a cleaned CSV file or perform data analysis.

## Materials and methods

### DairyComp data extraction

The data extraction process consists of two steps. First, a few so-called ‘items’ are created using the ALTER function in DC followed by a ‘protocol’ command to generate a CSV file. The protocol command can be stored in DC for repeated use. For users who are not familiar with the ALTER or SETUP options in DC, the full software reference guide is found in DC Reference Guide: https://dc-help.vas.com/ReferenceGuide/Home-DC305RefGuide.htm. Detailed instructions for extracting the raw data from DC are found in **Appendix A in S1 Appendix** or in the Documentation tab of https://connect.doit.wisc.edu/dairy-copilot/.

### DairyCoPilot data cleaning

The CSV file from DC containing all variable names in the first row is read into the R Shiny application. Source code for DairyCoPilot is available at https://github.com/walleser/dairy-comp. The so-called ‘events’ from DC are automatically renamed e.g. ’SCOURS’ to ’DIARRHEA’ and missing events are filtered out. Missing columns required for further analysis in DairyCoPilot are created as placeholders. Remarks and protocols from DC are combined for completeness.

The data are grouped by cow identification (ID), lactation number (LACT), and event and the event number is appended to the event label. The dates and remarks are converted from a wide to a long format and the dates and remarks are appended to the event label respectively. The date and remarks are converted back from a long to a wide format with the variable names in the format of event, order, and if it is a date or remark. Missing events that are required for further analysis e.g. the first milk fever date for all ID and LACT are created as placeholders. The resulting variables are converted to the appropriate variable type, such as numeric or factor, for further analysis.

Cows who have not started their first lactation are filtered out and columns are created based on number of occurrences of an event for all ID and LACT. Columns are renamed as shown in **Appendix B in S1 Appendix**. The date and remark of a ‘removed’ event are created based on the sold and died events. Observations are excluded if the birth date, fresh date, or first calving date are invalid or missing. Predefined variables are created based on cutoff days post-calving or based on a value for a given event as described in **Appendix B in S1 Appendix**, for example the ‘lactation group’ is 3 if the ‘lactation number’ is greater than or equal to 3, otherwise, the lactation group is the lactation number. Additionally, event occurrence, the days in milk of the first occurrence in the lactation period, and predefined variable based on cutoff days post calving for a given event are created as described in **Appendix B in S1 Appendix** e.g. ‘MLK_FVR< = 7’ is ‘1’ if the days in milk of the first occurrence of milk fever in the lactation period is less than or equal to 7 DIM, otherwise ‘MLK_FVR< = 7’ is ‘0’. Details regarding the cutoff value for the variables associated with transition cow health events are described in **Appendix B in S1 Appendix** and under the DairyCoPilot documentation tab. For binomial data, factor levels are reordered such that false or ‘0’ is used as reference level and true or ‘1’ as the alternative. For multinomial data, factor levels are reordered based on frequency from lowest to highest. For a given event, a rolling filter is applied such that if the next observation is recorded within a 3-day time lag of the previous observation, then only the previous observation is kept, else both observations are kept in the dataset. For example, if a cow has milk fever at 1 DIM, 2 DIM, and 4 DIM, then only the observations at 1 DIM and 4 DIM is kept. Detailed instructions for extracting the raw data from DC are found in **Appendix C in S1 Appendix**.

### Software requirements

All analyses are performed using R version 3.6.3 [6]. The R packages *readr*, *dplyr*, and *tidyr* through *tidyverse* are used for data importation, data manipulation, and data tidying respectively [7–10]. The R package *stringr* is used for working with strings, *forcats* for handling categorical variables including reordering character vectors to improve display, and *lubridate* for working with dates and times [11–13]. The R package *janitor* is used to generate two-way frequency tables for categorical analysis and *nnet* is used to fit multinomial log-linear models and to compute odds ratios [14, 15]. The R package *DT* provides an R interface to the JavaScript library *DataTables* used to create an interactive data table and *skimr* is used to provide summary statistics about variables in a data table [16–18]. The R package *reactable* is used to create an interactive data table and *reactablefmtr* is used to streamline and enhance the styling and formatting of tables [19, 20]. The R packages *ggplot2*, *GGally*, and *plotly* are used to create data visualizations, pairs plots, and interactive graphing respectively [21–23]. The R packages *shiny* and *shinydashboard* are used to build interactive web apps and dashboards [22, 24]. The DairyCoPilot Dashboard R Shiny application is published using RStudio Connect: https://connect.doit.wisc.edu/dairy-copilot/ [25]. Connect assigns a distinct temporary directory to every process it initiates. Interactive applications such as Shiny are granted write access to the directory where the unprocessed code is stored. This directory serves as the working directory when launching an application, and any data written to it is accessible exclusively to processes linked with that particular application and is not visible to processes linked to other content. The data in the application directory remains accessible until the application is redeployed in Connect, which generates a new application directory exclusively containing the newly deployed content.

## Results

The following sections describe the four tabs of the DairyCoPilot Dashboard.

### Input tab

**Fig 1:**
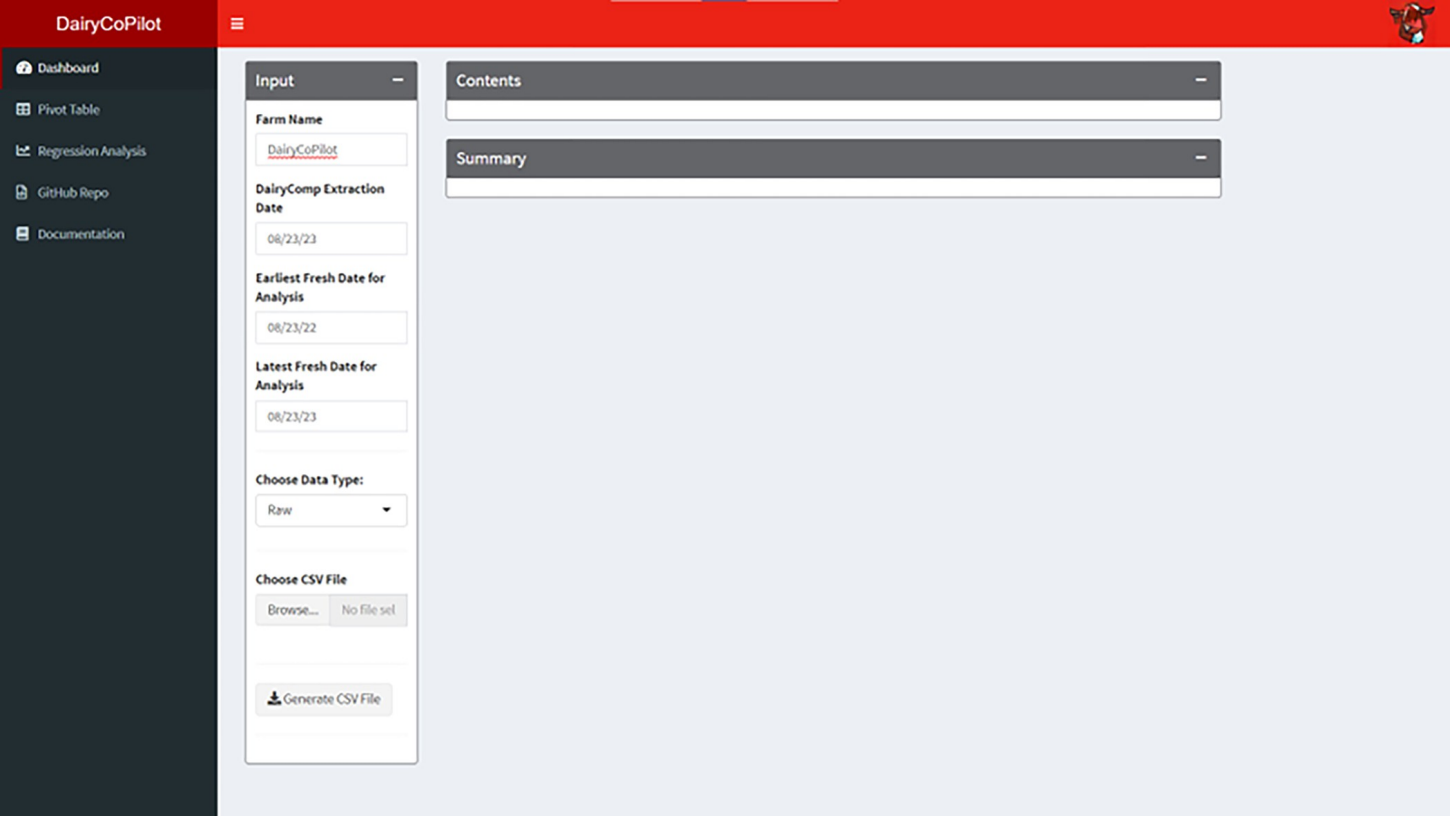
Dashboard Tab of the DairyCoPilot application before data upload.

**Fig 2:**
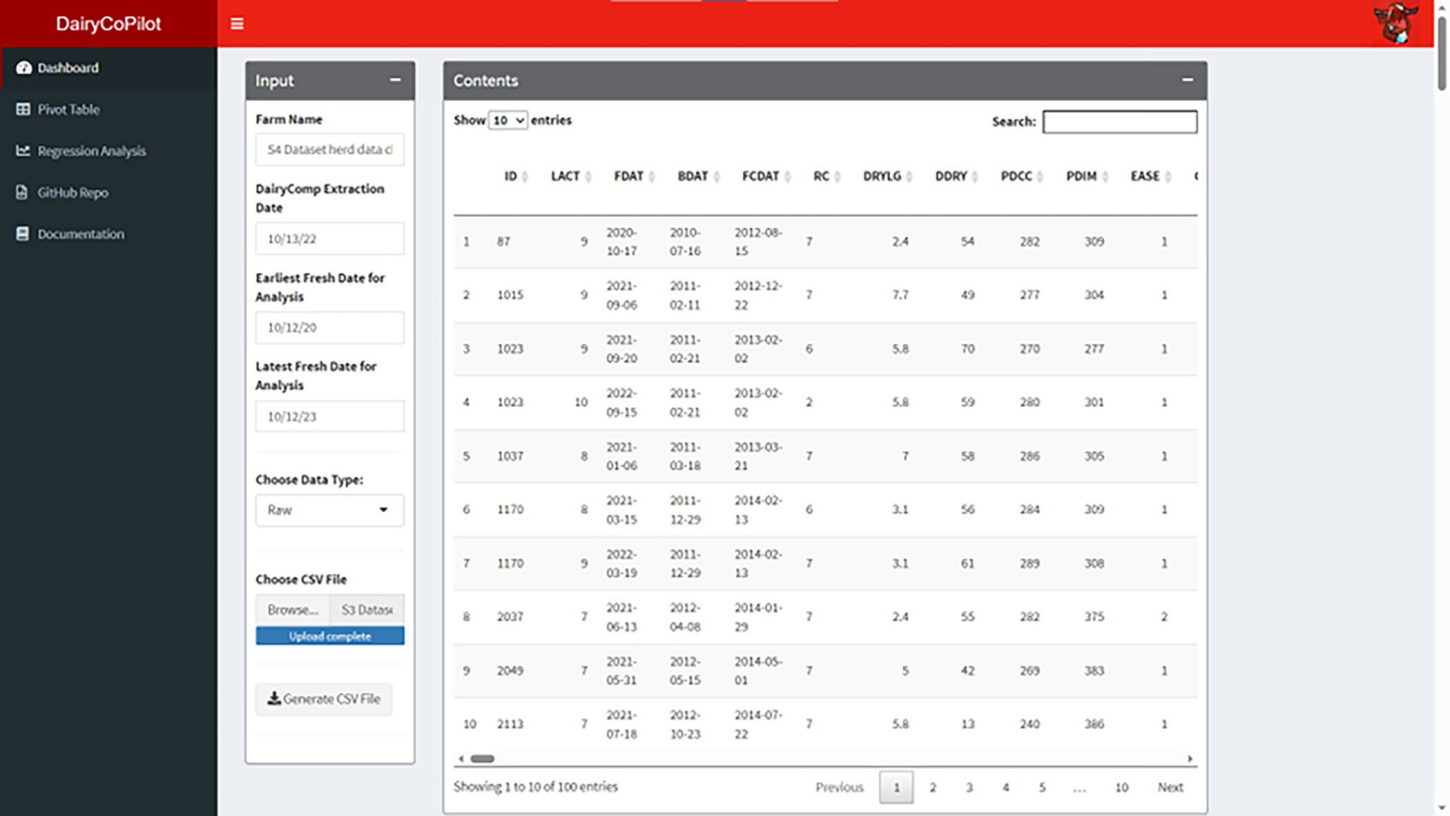
Dashboard Tab of the DairyCoPilot application after data upload. The user can input the farm name, the date of data extraction from DairyComp, and starting and ending fresh dates for inclusion in the data analysis. The Contents panel displays the transformed dataset and is searchable. The Summary panel includes information about all variables including descriptive statistics and missing variables.

The Dashboard tab of the DairyCoPilot R Shiny app contains three panels. The Input panel is used to upload the downloaded CSV file from DC (**Fig 1**). Moreover, users are able to input the farm name, DairyComp extraction date, and earliest fresh date for analysis, most recent fresh date for analysis. Users can upload a ‘raw’ or previously unprocessed CSV, download a ‘cleaned’ CSV, make edits if there are errors in the DC records, and save these changes. Users can upload a previously cleaned CSV using the download button in the app or edited CSV in a spreadsheet app e.g. Microsoft Excel if necessary. The Contents panel is used to display the first 100 rows of the dataset providing filtering, pagination, and sorting (**Fig 2**). The Summary panel is used to display summary statistics the user can skim to understand the data (**Fig 2**). Results are printed horizontally with a section for each variable type and a row for each variable.

### Pivot Table tab

**Fig 3:**
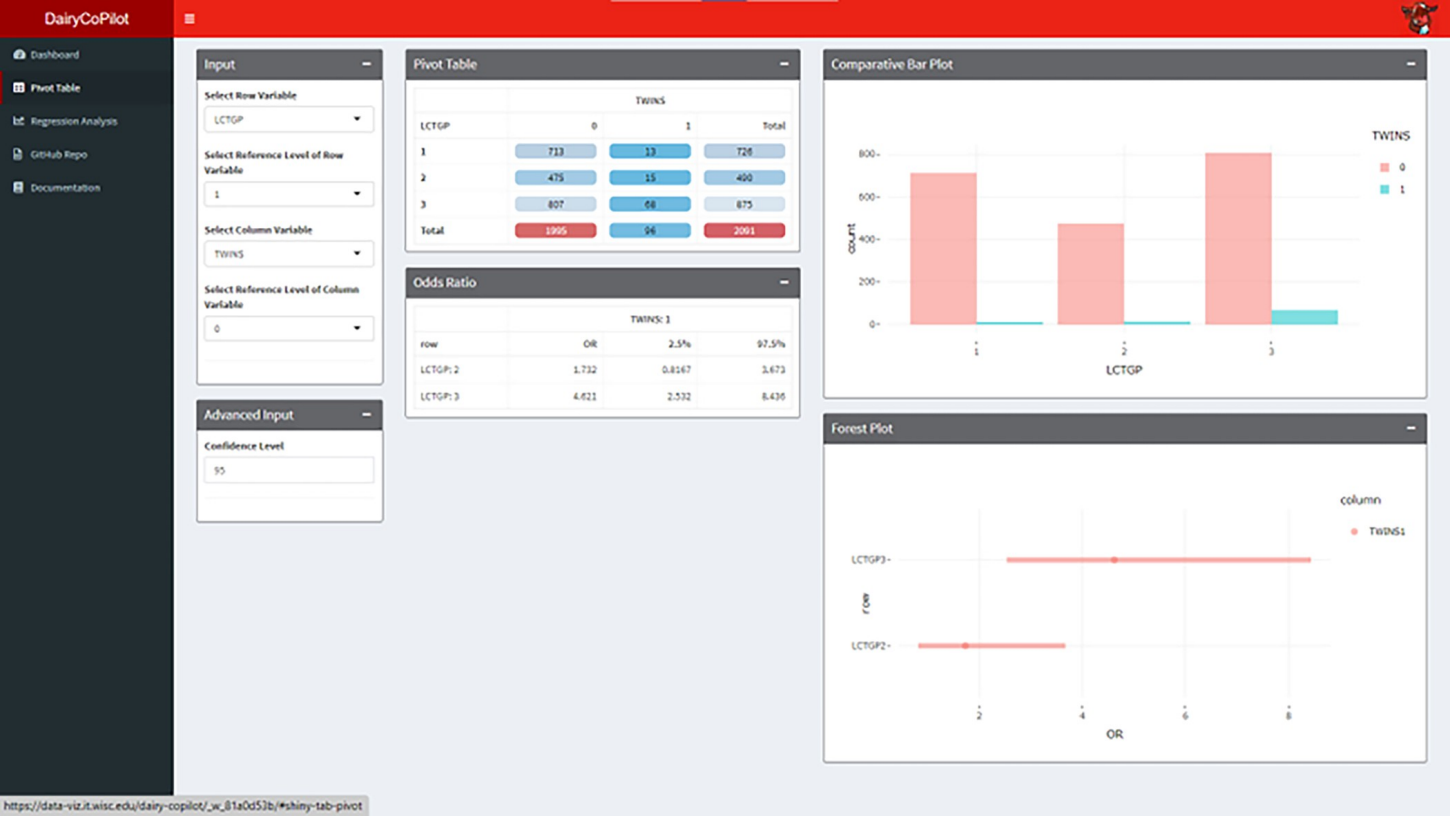
Pivot Table tab. The Pivot Table of the DairyCoPilot application allows the user to perform statistical analysis for categorical variables and visualize two-way associations. Odd ratios and 95% confidence intervals allow the user to quantify pairwise associations and assess the statistical significance.

The Pivot Table tab is used for categorical analysis of multinomial data (**Fig 3**). The tab contains three columns for a total of six panels. The first column is the Input panel that is used to select the row variable and column variable for two-way tabulation where the user can also specify the reference level of both variables for further use. The Advanced Input panel allows users to specify a confidence level different than the default 95% level. The second column includes the Pivot Table panel of a two-way frequency table with the row and column sums, where the depth of the color mapping corresponds to counts, such that blue is equivalent to low counts and red is equivalent to high counts. The second column also includes the Odds Ratio panel of the odds ratio (OR) and corresponding confidence interval (CI) for each alternative level with respect to the reference levels previously specified [26]. The OR and CI are computed by fitting a multinomial log-linear model via a neural network [14]. The third column is used to visualize the data where the Comparative Bar Plot panel is used to display the count data in the pivot table and the Forest Plot panel is used to present the point estimate and interval data in the OR table. All plots are interactive with details on demand and can be downloaded as PNG files.

### Regression Analysis tab

**Fig 4:**
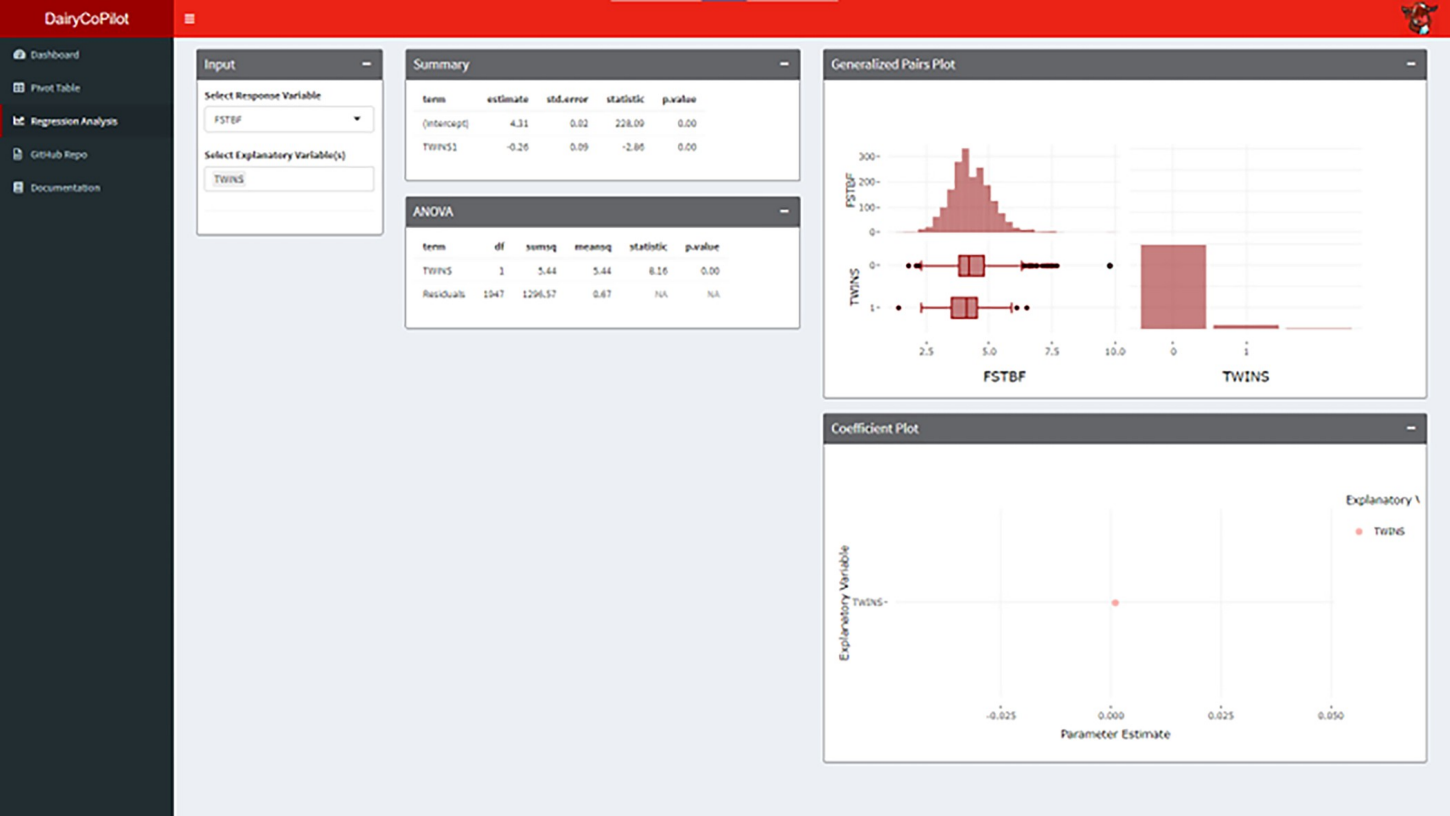
Regression Analysis tab. Regression Analysis in the DairyCoPilot application allows the user to perform linear and logistic regression. The user can select a response variable and multiple explanatory variables followed by different variables to control for confounding across groups. Graphical analysis in the Generalized Pairs Plot and Coefficient plot panels visualize the relationships between variables in the Summary and ANOVA panels.

The Regression Analysis tab is used to fit a regression model for an outcome variable of interest (**Fig 4**). Similar to the previous tab, the tab contains three columns for a total of five panels. The first column is the Input panel that is used to select the response and explanatory variables. The R Shiny app automatically recognizes if the response variable is a numeric or categorical variable as well as if the model is linear or logistic regression respectively [14, 27–29]. The second column includes the Summary panel that is the coefficients table for the regression model with estimate for slope, standard error, t-statistic, and p-value for each term in the regression model. The table can be used to determine the adjusted effect of each explanatory variable on the response variable and the statistical significance of the association. The second column also includes the ANOVA panel, that is the Analysis of Variance table of the regression model described above with degrees of freedom, sum of squared errors, mean squared errors, F-statistic, and p-value for each term in the regression model [26]. The ANOVA table can be used to determine the variation of the response variable due to variation in the explanatory variable and the statistical significance of the association. The third column is used to visualize the data where the Generalized Pairs Plot panel automatically displays a comparative plot for each pair of variables in the regression model, depending on the type of data. The Coefficient Plot is used to visualize the estimate and 95% CI using the coefficient table. All plots are interactive with details on demand and can be downloaded as PNG files.

### Documentation tab

**Fig 5:**
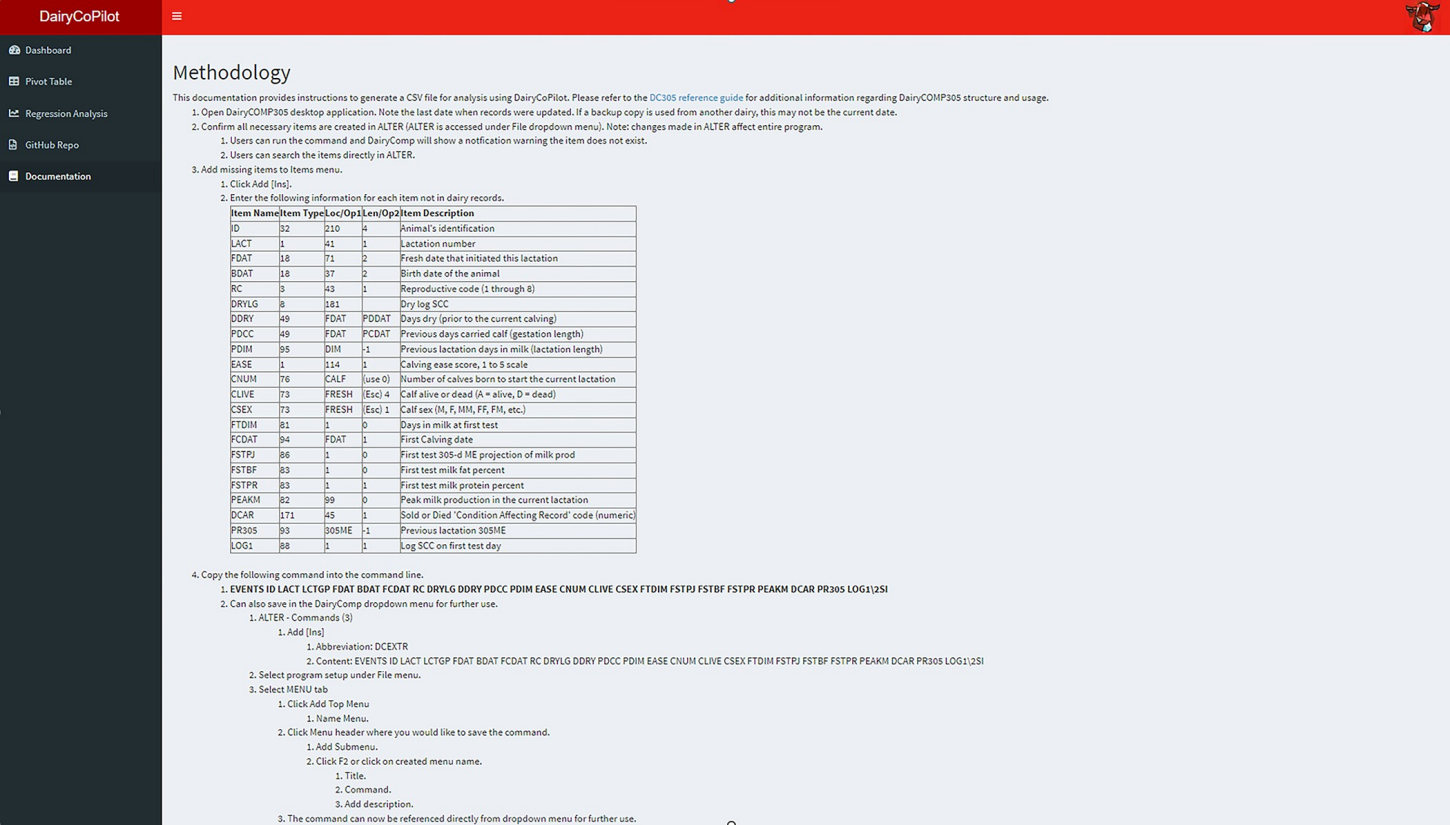
Documentation page. Documentation in the DairyCoPilot application provides instructions to generate a CSV file for analysis using DairyCoPilot.

The Documentation tab provides instructions to generate the input CSV file in DC that is used for the data cleaning step by DairyCoPilot (**Fig 5**). A glossary of variables is provided for variables used to generate the output CSV file for the analysis in DairyCoPilot. Finally, an example dataset is provided for download for interested users to try out and test the app.

### Software usage and example

A case study is performed to demonstrate the potential uses of the DairyCoPilot R Shiny app. A 1000-cow dairy in Wisconsin, USA provided access to DC records collected in 2022. Following data upload, summary statistics and cow records are displayed (**Fig 2**). Transformed data can be downloaded directly as a CSV file and additional analysis is performed using the Pivot Table and Regression Analysis tabs.

In the Pivot Table tab, the input selected for the Row variable is LCTGP with a reference level of 1 and the column variable is REMVD with a reference level of 0. The Pivot Table panel cross-tabulates categorical variables by level (**Fig 3**). In the LCTGP by REMVD example, the three lactation groups are split into 1st, 2nd, and 3rd+ lactations as rows in the panel and removed status (1) or not removed (0) as columns. The Odds Ratio panel provides a measure of association between two categorical risk factors. The odds of removal as a second lactation cow compared to a first lactation cow is 1.2 (95% CI: 0.9–1.8) indicating no difference in removal between first and second lactation at a 95% confidence level. The 3+ cows have a greater odds of removal than first lactation animals OR: 2.8 (95% CI: 1.7–3.1). The pivot table is visualized in a Comparative Barplot panel for total counts of removed and non-removed by lactation and OR is represented in the Forest Plot panel. Both of these graphs provide summary statistics by hovering over the image. Additionally, the graphics can be downloaded as an image file.

The Regression Analysis tab is used to quantify the associations between first-test butterfat percent (FSTBF), retained placenta (RP), lactation (LACT), and twins (TWIN). The summary panel provides estimates for regression coefficients and statistical significance (**Fig 4**). FSTBF is not associated with lactation (estimate: 0.0; p-value: 0.96), negatively associated with twins (estimate: -0.2; p-value: 0.02), and FSTBF is negatively associated with the occurrence of retained placenta (estimate: -0.3; p-value: 0.02). Similar interpretations are generated using the ANOVA table. The Generalized Pairs Plot panel presents a representation of associations between coefficients. The Coefficient Plot panel provides a visualization of the regression parameter estimates. Both of these graphs provide summary statistics by hovering over the image. Additionally, the graphics can be downloaded as an image file.

Any choices of analysis for association of risk factors and outcome variables can be quantified using the DairyCoPilot tool, except for associations where limited numbers of observations per category result in non-convergence of the regression models. For such cases, the Pivot Table tab is preferred.

### Example analysis

An example analysis is completed to demonstrate a basic analysis of a dairy herd and can be replicated by downloading the example.csv found in the documentation tab. The CSV file is included in the supplementary materials **S1 Dataset herd data.csv**. The CSV file was extracted from DairyComp on 10/13/22, and earliest fresh date for analysis was selected 10/12/20 to capture 2 years of date and last date was selected as 10/12/23. The data included 2106 rows. The file was then downloaded as a CSV file for storage and availability outside of DairyCoPilot and is included as **S2 Dataset herd data cleaned.csv**. As an example, the relationship between twins, health, and milk production were examined using the Pivot Table tab. The association between twins (TWINS) and lactation group (LCTGP) were quantified and visualized. The reference level for LCTGP was selected as lactation 1 and for TWINS as 0. The OR for TWINS between LCTGP 1 and 2 was 1.7 (95% CI: 0.8–3.7) and LCTGP 1 and 3 was 4.6 (95% CI: 2.5–8.4). The OR between removal from the herd before 60 DIM and twins was 3.4 (95% CI: 1.9–6.2). The numerical variables and multiple variable model were inspected the Regression Analysis tab. First test butterfat was selected as an outcome variable for this example. The effect of Twins as an explanatory value for FSTBF was added as an explanatory variable. The estimated effect of twins on FSTBF was -0.3 with a p-value less than 0.01. In the categorical analysis the association of LCTGP and TWINS was noted. Therefore, an additional explanatory variable was added to the regression equation for FSTBF = TWINS + LCTGP. The resulting estimate for FSTBF changed to -0.28 with a p-value less than 0.01. Finally, ketosis (KET) was added to the regression equation resulting in FSTBF = TWINS + LCTGP + KET. The p-value for KET was 0.48 and the user may elect to remove it from the regression model. Multiple other factors can be added to the regression model to improve user understanding and prediction performance. The resulting evaluation shows that twins are associated with lower FSTBF when controlling for LACT at this dairy.

## Discussion

### Dairy data collection

A significant proportion of dairy data is manually acquired and recorded. Events such as disease diagnosis and subsequent recording are subject to human error. Errors in data recording will propagate forward into subsequent analysis. The DairyCoPilot tool provides a method for reducing time to analysis but cannot improve the accuracy of input data. However, it is user responsibility to correctly validate data before data analysis.

### Data analysis tools

Agricultural production is a data-rich environment that requires advanced analysis for informed decision-making processes [30, 31]. Advanced training can be accomplished through outreach and extension services with fit-for-purpose tools that reduce barriers of application by end users. Training and analysis tools such as DairyCoPilot presented in this study, fill a need in the dairy industry that is currently unmet. Decision-making processes using graphic analysis or trend observation alone are not powerful enough for modern production systems [32, 33]. More advanced multiple variable statistical tools allow users to control multiple confounding variables such as lactation or milk production simultaneously [34]. Data analysis tools used for local on-farm decision support should be customizable to user demands. The proposed DairyCoPilot application allows for customization of analysis choices and guarantees data ownership resulting in a locally secure tool.

Platforms providing data analysis including visualization, dashboarding, and economic evaluation of cow health and production include milking services companies e.g. Lely T4C, DeLaval DelPro, etc.; herd management platforms e.g. DC, animal, Bovisync, Dairy Data Wearhouse, etc.; and cow monitoring technologies e.g. SmaXtec, Connectera, CowManager, etc. [5, 35–41]. Tools for exporting graphs and reports are limited, and statistical data analysis can be absent from these applications. Licenses must be paid, or large purchases must be made from these companies in order to access the data analysis tools. The DairyCoPilot application has advantages for data assets used for on-farm decision-making processes, because DairyCoPilot is free, easy-to-use, secure, and customizable.

### Big data approaches

The alternative to modular data analysis tools is a big data analysis approach. These analysis systems take in data from multiple diverse sources, compile them, and generate inferences. For example, Dairy Data Warehouse provides integrated data storage, analytics, and forecasting for enrolled dairies [41]. These services are billed on a per-cow subscription fee. Another example of a big data approach is the UW Madison Dairy Brain and Data Hub [42].

These tools enhance data analysis on farms. Questions regarding data ownership and security are important considerations regarding these tools [43, 44]. Food production stakeholders, including commercial and governmental organizations, will need to reach agreements about data sharing, ownership, and management. These challenges indicate that small data approaches, such as the proposed DairyCoPilot application, are still a viable option that allows improved and customized control by individual end users. Small data applications can be integrated into big data applications in the future.

### Strengths

First and foremost, DC does not provide advanced data analysis required for dairies to gain insights and make data-driven decisions for complex problems. The user interface is extremely basic and customization is insufficient where the software is only developed for Windows desktops. The management application has a limited set of features lacking the advanced functionality to manipulate data, create graphics, and generate figures and tables for estimation and prediction.

Conversely, DairyCoPilot provides statistical tools for both categorical data analysis and regression analysis. The program provides an elegant and powerful web framework where the user interface is dynamic to conditionally generate input controls and uses reactive programming to automatically update outputs when inputs change. The web application can clean data for downstream data analysis, create interactive graphics for exploratory and expository visualization, and export high quality figures using interactive views for documents, reports, and presentations. Lastly, DairyCoPilot is mobile-friendly, desktop progressive web application for all device platforms.

### Limitations

Currently, DairyCoPilot only handles a subset of events and items as detailed in **Appendix B in S1 Appendix**. The program is limited to analysis of cow data and is not yet available for calf data but can be added in the future. The implementation of software requires appropriate statistical training which must be obtained separately from the supplied software tool. Consultants must still use a holistic approach to evaluating dairies in addition to statistical tools where visual appraisal of dairies can provide information not contained in records and can even contradict records. The main drawback of the data analysis tool is slow performance for extremely large data sets containing multiple recordings of events compared to other languages. Accordingly, the lack of scalability compared to more popular frameworks such as Node.js or React can be an issue for dairies that need to handle a lot of traffic or requests. Currently, the regression tab does not provide the option to add random effects in the model. Additionally, the application does not provide the functionality to check the assumptions of statistical methods e.g. diagnostic plots for linear models and generalized linear models. This will be available in forthcoming updates.

## Conclusions and outlook

Tools for automated data analysis are necessary for training the next generation of food animal consultants and life scientists. Tools, such as DairyCoPilot, help shift the burden of data formatting and editing in terms of time and effort to highly trained individuals. The food animal consultant does not need to be a statistical expert if automated statistical analysis tools are accessible and if such analysis is part of their daily discussion and decisions.

The outlook for local data analysis tools such as the DairyCoPilot is the expansion of variable formatting into customized categorical variables, interaction terms and random effects models in the near future. DairyCoPilot is the first step in developing advanced freely available tools for rapid analysis of records from dairy farms that are translatable to all other animal species and healthcare settings. Additionally, adoption of these tools will facilitate commercial providers, such as VAS, to include advanced graphical and statistical tools directly into their software updates and to improve workflows for data extraction to support other tools. The DairyCoPilot application represents an important step in turning farm records into data assets for more customized decision-making processes by informed consultants in the life sciences.

## Supporting information

S1 Appendix Additional DairyComp extraction information. (DOCX)

S1 Dataset Dataset herd data. Herd data Raw CSV extracted from DairyComp.
(CSV)

S2 Dataset Dataset herd data cleaned. Herd data cleaned CSV data using DairyCoPilot tool.
(CSV)
