# Supplementary material for: DairyCoPilot—Automated data compilation and analysis tools for DairyComp data assets
Source: PLoS One. 2024 Apr 18;19(4):e0297827. doi: 10.1371/journal.pone.0297827 (PMC11025776; doi:10.1371/journal.pone.0297827)
Supplement: S1 Appendix — (DOCX) [file pone.0297827.s001.docx]

**S1 Appendix A**

**Instructions:**

1. Open DairyComp desktop software. Make a note of the last date records were updated. If using a backup copy from another dairy this may not be the current date.
2. Ensure all necessary items are created in ALTER (ALTER is accessed under File dropdown menu):

CAUTION: Changes made in ALTER affect the entire program, be cautious

1. Users can run the following command and DairyComp will warn their item does not exist
2. Items can be searched directly in ALTER
3. Add missing items to Items menu
   1. Click Add [Ins]
   2. Enter information in the chart below for each item not found in dairy records

| **Item Name** | **Item Type** | **Loc/Op1** | **Len/Op2** | **Item Description** |
| --- | --- | --- | --- | --- |
| ID | 32 | 210 | 4 | Animal's identification |
| LACT | 1 | 41 | 1 | Lactation number |
| FDAT | 18 | 71 | 2 | Fresh date that initiated this lactation |
| BDAT | 18 | 37 | 2 | Birth date of the animal |
| RC | 3 | 43 | 1 | Reproductive code (1 through 8) |
| DRYLG | 8 | 181 |  | Dry log SCC |
| DDRY | 49 | FDAT | PDDAT | Days dry (prior to the current calving) |
| PDCC | 49 | FDAT | PCDAT | Previous days carried calf (gestation length) |
| PDIM | 95 | DIM | -1 | Previous lactation days in milk (lactation length) |
| EASE | 1 | 114 | 1 | Calving ease score, 1 to 5 scale |
| CNUM | 76 | CALF | (use 0) | Number of calves born to start the current lactation |
| CLIVE | 73 | FRESH | (Esc) 4 | Calf alive or dead (A = alive, D = dead) |
| CSEX | 73 | FRESH | (Esc) 1 | Calf sex (M, F, MM, FF, FM, etc.) |
| FTDIM | 81 | 1 | 0 | Days in milk at first test |
| FCDAT | 94 | FDAT | 1 | First Calving date |
| FSTPJ | 86 | 1 | 1 | First test 305-d ME projection of milk prod |
| FSTBF | 83 | 1 | 0 | First test milk fat percent |
| FSTPR | 83 | 1 | 1 | First test milk protein percent |
| PEAKM | 82 | 99 | 0 | Peak milk production in the current lactation |
| DCAR | 171 | 45 | 1 | Sold or Died "Condition Affecting Record" code (numeric) |
| PR305 | 93 | 305ME | -1 | Previous lactation 305ME |
| LOG1 | 88 | 1 | 1 | Log SCC on first test day |
| Table 1:  Items to be created in the DairyComp file for the dairy to be analyzed. | | | | |

1. Copy the following command into the command line:
   1. **EVENTS ID LACT LCTGP FDAT BDAT FCDAT RC DRYLG DDRY PDCC PDIM EASE CNUM CLIVE CSEX FTDIM FSTPJ FSTBF FSTPR PEAKM DCAR PR305 LOG1\2SI**
   2. This command can also be saved in the DairyComp dropdown menu for future use:
      1. ALTER – Commands (3)
         1. Add [Ins]
            1. Abbreviation: DCEXTR
            2. Content: EVENTS ID LACT LCTGP FDAT BDAT FCDAT RC DRYLG DDRY PDCC PDIM EASE CNUM CLIVE CSEX FTDIM FSTPJ FSTBF FSTPR PEAKM DCAR PR305 LOG1\2SI
      2. Under File select program setup
      3. Select MENU tab
         1. Click Add Top Menu
            1. Name menu
         2. Click Menu header you would like command to be saved under
            1. Add Submenu
            2. Click F2 or click in created menu name

Title

Command (DCEXTR)

Add description

- - - 1. The Command can now be referenced directly from dropdown menu in the future

1. Select a date 2-2.5 years prior to DC date
2. Select Last date you wish to analyze data for
3. Select Events
   1. Select All will contain the most information and is preferred
   2. Can individually choose Events as well
      1. MUST include FRESH
   3. Select optional REM pattern:
      1. Select default “none” by clicking “OK”
   4. This may take a few minutes
4. Write data to CSV file
   1. Find floppy disk icon above command line
   2. Click downward facing triangle to the right
   3. Save as CSV (Do Not save as XLS)
   4. Choose location and name for CSV
5. The downloaded CSV is now ready to be analyzed using DairyCoPilot app

**S1 Appendix B**

| **Item Name** | **Item Description** |
| --- | --- |
| ID | Animal's identification |
| LACT | Lactation number |
| LCTGP | Lactation groups (1: lactation number is 1; lactation number is 2; 3: lactation number is greater than or equal to 3) |
| FDAT | Fresh date that initiated this lactation |
| BDAT | Birth date of the animal |
| TOCU1 | Date of last move to close-up pen |
| TOCU2 | Date of previous move to close-up pen |
| RC | Reproductive code (1 through 8) |
| DRYLG | Dry log SCC |
| DDRY | Days dry (prior to the current calving) |
| PDCC | Previous days carried calf (gestation length) |
| PDIM | Previous lactation days in milk (lactation length) |
| EASE | Calving ease score, 1 to 5 scale |
| CNUM | Number of calves born to start the current lactation |
| CLIVE | Calf alive or dead (A: alive; D: dead) |
| CSEX | Calf sex (M, F, MM, FF, FM, etc.) |
| FTDIM | Days in milk at first test |
| FSTPJ | First test 305-d ME projection of milk prod |
| FSTBF | First test milk fat percent |
| FSTPR | First test milk protein percent |
| PEAKM | Peak milk production in the current lactation |
| DCAR | Sold or Died "Condition Affecting Record" code (numeric) |
| DATS | Date sold |
| REMS | Remark of the sold event |
| DATD | Date died |
| REMD | Remark of the died event |
| 1DMF | Date of 1st milk fever in the current lactation |
| REMMF | Remark of 1st milk fever in the current lactation |
| XMF | Count of all milk fever events in the current lactation |
| 1DRP | Date of 1st retained placenta in the current lactation |
| REMRP | Remark of 1st retained placenta in the current lactation |
| XRP | Count of all RP events in the current lactation |
| 1DME | Date of 1st metritis event in the current lactation |
| REMME | Remark of 1st metritis event in the current lactation |
| XMETR | Count of all metritis events in the current lactation |
| 1DKE | Date of 1st ketosis event in the current lactation |
| REMKE | Remark of 1st ketosis event in the current lactation |
| XKET | Count of all ketosis events in the current lactation |
| 1DDA | Date of 1st DA event in the current lactation |
| REMDA | Remark of 1st DA event in the current lactation |
| XDA | Count of all DA events in the current lactation |
| 1DPN | Date of 1st pneumonia event in the current lactation |
| REMPN | Remark of pneumonia event in the current lactation |
| XPNEU | Count of all pneumonia events in the current lactation |
| 1DDIA | Date of 1st diarrhea event in the current lactation |
| REMDI | Remark of 1st diarrhea event in the current lactation |
| XDIAR | Count of all diarrhea events in the current lactation |
| 1DLAM | Date of 1st lame event in the current lactation |
| REMLM | Remark of 1st lame event in the current lactation |
| XLAME | Count of all lame events in the current lactation |
| 1DFT | Date of 1st foot trim event in the current lactation |
| REMFT | Remark of 1st foot trim event in the current lactation |
| XFTRM | Count of all foot trim events in the current lactation |
| 1DMA | Date of 1st mastitis event in the current lactation |
| REMMA | Remark of 1st mastitis event in the current lactation |
| XMAST | Count of all mastitis events in the current lactation |
| 1DIL | Date of 1st ILLMISC event in the current lactation |
| REMIL | Remark of 1st ILLMISC event in the current lactation |
| XILL | Count of all ILLMISC events in the current lactation |
| DATS | Date removed |
| REMS | Remark of the removed event |
| FRESH_MONTH | Fresh date that initiated this lactation (YYYY-MM) |
| DIM | Days in milk (difference between fresh date and data extraction date or date of removal if removed) |
| FRESH 372 TO 7 | Days in milk between 7 and 372 |
| FRESH 386 TO 21 | Days in milk between 21 and 386 |
| FRESH 425 TO 60 | Days in milk between 60 and 425 |
| BIRTH_DATE | Birth date of the animal |
| AGE_AT_CALVING_(MONTHS) | Age at calving (from birth date to fresh date; months) |
| AGE_AT_CALVING_1ST_LACT_(MONTHS) | Age at calving (from birth date to first calving date; months) |
| ABORT | Abort (1: gestation length less than 260; 0: otherwise) |
| CALVING_EASE_>=2 | Calving ease score greater than or equal to 2 |
| CALVING_EASE_>=3 | Calving ease score greater than or equal to 3 |
| TWINS | Twins (1: number of calves born to start the current lactation greater than 1; 0: number of calves born to start the current lactation equal to 1) |
| STILLBIRTH | Stillborn (1: dead; 0: otherwise) |
| CALF_SEX | Calf sex |
| MALE_CALF | Male calf |
| FPR | Fat protein ratio |
| FPR>1.4 | Fat protein ratio greater than 1.4 (1: fat protein ratio greater than or equal to 1.4; 0: otherwise) (Duffield et al., 1997) |
| SOLD | Sold |
| SOLD_DIM | Days in milk at sale |
| SOLD<=60 | Days in milk at sale less than or equal to 60 (1: sale less than or equal to 60; 0: otherwise) |
| DIED | Died |
| DIED_DIM | Days in milk at death |
| DIED<=60 | Days in milk at death less than or equal to 60 (1: death less than or equal to 60; 0: otherwise) |
| REMVD | Removed |
| REMVD_DIM | Days in milk at removal |
| REMVD<=60 | Days in milk at removal less than or equal to 60 (1: removal less than or equal to 60; 0: otherwise) |
| MLK_FVR | Milk Fever |
| MLK_FVR_DIM | Days in milk at 1st milk fever event |
| MLK_FVR<=7 | Days in milk at 1st milk fever event less than or equal to 7 (1: days in milk less than or equal to 7; 0: otherwise) |
| RP | Retained placenta |
| RP_DIM | Days in milk at 1st retained placenta event |
| RP<=7 | Days in milk at 1st retained placenta event less than or equal to 7 (1: days in milk less than or equal to 7; 0: otherwise) |
| METR | Metritis |
| METR_DIM | Days in milk at 1st metritis event |
| METR<=21 | Days in milk at 1st metritis event less than or equal to 21 (1: days in milk less than or equal to 21; 0: otherwise) |
| KET | Ketosis |
| KET_DIM | Days in milk at 1st ketosis event |
| KET<=60 | Days in milk at 1st ketosis event less than or equal to 60 (1: days in milk less than or equal to 60; 0: otherwise) |
| DA | DA |
| DA_DIM | Days in milk at 1st DA event |
| DA<=60 | Days in milk at 1st DA event less than or equal to 60 (1: days in milk less than or equal to 60; 0: otherwise) |
| PNEU | Pneumonia |
| PNEU_DIM | Days in milk at 1st pneumonia event |
| PNEU<=60 | Days in milk at 1st pneumonia event less than or equal to 60 (1: days in milk less than or equal to 60; 0: otherwise) |
| DIARR | Diarrhea |
| DIARR_DIM | Days in milk at 1st diarrhea event |
| DIARR<=60 | Days in milk at 1st diarrhea event less than or equal to 60 (1: days in milk less than or equal to 60; 0: otherwise) |
| LAME | Lameness |
| LAME_DIM | Days in milk at 1st lameness event |
| LAME<=60 | Days in milk at 1st lameness event less than or equal to 60 (1: days in milk less than or equal to 60; 0: otherwise) |
| FTRM | Foot trim |
| FTRM_DIM | Days in milk at 1st foot trim event |
| FTRM<=60 | Days in milk at 1st foot trim event less than or equal to 60 (1: days in milk less than or equal to 60; 0: otherwise) |
| MAST | Mastitis |
| MAST_DIM | Days in milk at 1st mastitis event |
| MAST<=60 | Days in milk at 1st mastitis event less than or equal to 60 (1: days in milk less than or equal to 60; 0: otherwise) |
| ILLMISC | Miscellaneous illness |
| ILLMISC_DIM | Days in milk at 1st miscellaneous illness event |
| ILLMISC<=60 | Days in milk at 1st miscellaneous illness event less than or equal to 60 (1: days in milk less than or equal to 60; 0: otherwise) |
| Cutoff values were based high-risk time periods defined by previous research and opinion: immediately after calving (<=7), during the transition period (<=21), and during early portion of lactation (<=6) (Drackley, 1999; McArt et al., 2012; Barragan et al., 2018; McArt and Neves, 2020) | |

**S1 Appendix C**

**Instructions:**

1. Event dates are filtered between the earliest and latest fresh date for analysis
2. Observations are grouped by animal ID, lactation number, and event. Observations are arranged by event date in animal ID, lactation number, and event. Event dates are filtered where the next observation is removed if it is within a 3-day time lag of the previous observation. Observations are ungrouped.
3. Event names are modified by case when

| **Match Event Name** | **Replacement Event Name** |
| --- | --- |
| FRESH | FRESH |
| ABORT | ABORT |
| SOLD | SOLD |
| DIED | DIED |
| MF | MF |
| RP | RP |
| METR | METR |
| KETOSIS | KETOSIS |
| DA | DA |
| LDA | DA |
| RDE | DA |
| PNEU | PNEU |
| SCOURS | DIARRHEA |
| LAME | LAME |
| FOOTRIM | FOOTRIM |
| ILLMISC | ILLMISC |
| RESP | PNEU |
| PNEUMON | PNEU |
| MFEVER | MF |
| MILKFEV | MF |
| MILKFVR | MF |
| MLKFEVR | MF |
| MLKFVR | MF |
| RETAINP | RP |
| MAST | MAST |
| MET | METR |
| METRITIS | METR |
| KET | KETOSIS |
| DIARRH | DIARRHEA |
| DIARHEA | DIARRHEA |
| HOOFROT | LAME |
| FOOTROT | LAME |
| FOOTRMK | FOOTRIM |
| TRIM | FOOTRIM |
| ILL | ILLMISC |

Otherwise, event names default to as is. Missing event names are removed.

1. Modify FTDIM, FSTPJ, FSTBF, FSTPR, PEAKM, DCAR, and PR305 if value is ‘0’, then replace with ‘NA’. Otherwise, defaults to as is.
2. Modify LOG1 if FSTPJ is ‘NA’, then LOG1 is ‘NA’. Otherwise, LOG1 defaults to as is.
3. Add ID, LACT, FDAT, BDAT, FCDAT, RC, DRYLG, DDRY, PDCC, PDIM, EASE, CNUM, CLIVE, CSEX, FTDIM, FSTPJ, FSTBF, FSTPR, PEAKM, DCAR, PR305, LOG1, Event, Date, Remark, Protocols, and Technician as placeholders if missing where all values are ‘NA’.
4. Keep ID, LACT, FDAT, BDAT, FCDAT, RC, DRYLG, DDRY, PDCC, PDIM, EASE, CNUM, CLIVE, CSEX, FTDIM, FSTPJ, FSTBF, FSTPR, PEAKM, DCAR, PR305, LOG1, Event, Date, Remark, Protocols, and Technician. Drop all other variables.
5. Modify Remark where Remark, Protocols, and Technician are concatenated. Drop Protocols and Technician.
6. Event names are filtered to keep FRESH, ABORT, SOLD, DIED, MF, RP, METR, KETOSIS, DA, PNEU, DIARRHEA, LAME, FOOTRIM, MAST, and ILLMISC. Drop all other observations.
7. Observations are grouped by animal ID, lactation number, and event. Modify event names where event name and event number are concatenated. Observations are ungrouped.
8. Pivot data from wide to long format by Date and Remark. Modify event names where event name and if it is a Date or Remark are concatenated. Pivot data from long to wide format by event name.
9. Add MF_1_Date, RP_1_Date, METR_1_Date, KETOSIS_1_Date, PNEU_1_Date, DIARRHEA_1_Date, LAME_1_Date, FOOTRIM_1_Date, MAST_1_Date, and ILLMISC_1_Date as placeholders if missing where all values are ‘NA’.
10. Lactation number is filtered to keep all LACT greater than 0. Drop all other observations. Fresh dates are filtered between the earliest and latest fresh date for analysis. Dates are transformed to Date objects. Remarks are transformed to character objects.
11. Observations are grouped by row. Create XMF, XRP, XMETR, XKET, XDA, XPNEU, XDIAR, XLAME, XFTRM, XMAST, and XILL to count the number of non-missing events for each animal ID and lactation number. Observations are ungrouped.
12. Create DATS as SOLD_1_Date, REMS as SOLD_1_Remark, DATD as DIED_1_Date, and REMD as DIED_1_Remark. Create DATR and REMR where if DATS is not missing, then DATR is DATS and REMR is REMS; else if DATD is not missing, then DATR is DATD and REMR is REMD. Otherwise, DATR and REMR are ‘NA’.
13. Birth date and fresh are filtered to keep strings between 8 to 10 characters for valid dates. Modify FCDAT if string is not between 8 to 10 characters, then replace with ‘NA’. Otherwise, FCDAT defaults to as is.
14. ID and RC are transformed to factor objects. BDAT, FDAT, FCDAT are transformed to Date objects. CLIVE and CSEX are transformed to character objects. LACT, FTDIM, FSTPJ, FSTBF, FSTPR, PEAKM, DCAR, PR305, DRYLG, LOG1, LACT, DDRY, PDCC, PDIM, EASE, and CNUM are transformed to numeric objects.
15. Create LACTGP where if LACT is greater than or equal to three, then LACTGP is ‘3’. Otherwise, LACTGP is LACT. Create FRESH_MONTH by formatting FDAT in ISO8601 character format and represent with year and month format. Create DIM where if DATR is missing, then DIM is the difference between DC extraction date and fresh date. Otherwise, DIM is the difference between date removed and fresh date.
16. Create FRESH 372 TO 7, FRESH 386 TO 21, and FRESH 425 TO 60 where if DIM is between 7 and 372, then FRESH 372 TO 7 is ‘1’; if DIM is between 21 and 386, then FRESH 386 TO 21 is ‘1’; and if DIM is between 60 and 425, then FRESH 425 TO 60 is ‘1’ respectively. Otherwise, the FRESH 372 TO 7, FRESH 386 TO 21, and FRESH 425 TO 60 are ‘0’.
17. Create BIRTH_DATE as BDAT. Create AGE_AT_CALVING_(MONTHS) as the number of months between BIRTH_DATE and FDAT. Create AGE_AT_CALVING_1ST_LACT_(MONTHS) as the number of months between BIRTH_DATE and FCDAT.
18. Create DDRY where if LACT is not equal to one, then DDRY is ‘NA’. Otherwise, DDRY defaults to as is. Create ABORT where if PDCC less than 260, then ABORT is ‘1’. Otherwise, ABORT is ‘0’. Create PDIM where if LACT is not equal to one, then PDIM is ‘NA’. Otherwise, PDIM defaults to as is.
19. Create CALVING_EASE_>=2 and CALVING_EASE_>=3 where if EASE is greater than or equal to two, then CALVING_EASE_>=2 is ‘1’ and if EASE is greater than or equal to three, then CALVING_EASE_>=3 is ‘1’. Otherwise, CALVING_EASE_>=2 and CALVING_EASE_>=3 are ‘0’.
20. Modify CNUM if CNUM is equal to one and EASE is not equal to zero, then CNUM is ‘NA’. Otherwise, CNUM defaults to as is. Modify CSEX if CNUM is missing, then CSEX is ‘NA’. Otherwise, CSEX defaults to as is. Modify CLIVE if CNUM is missing, then CLIVE is ‘NA’. Otherwise, CLIVE defaults to as is.
21. Create TWINS where if CNUM is greater than one, then TWINS is ‘1’ and CNUM is equal to one, then TWINS is ‘0’. Otherwise, TWINS is ‘NA’. Create STILLBIRTH where if any element of the string CLIVE matches the pattern ‘D’, then STILLBIRTH is ‘1’. Otherwise, STILLBIRTH is ‘0’. Create CALF_SEX as CSEX. Create MALE_CALF where if any element of the string CSEX matches the pattern ‘M’, then MALE_CALF is ‘1’. Otherwise, MALE_CALF is ‘0’.
22. Create FPR where it is the ratio between FSTBF and FSTPR. Create FPR>1.4 where if FPR is greater than 1.4, then FPR>1.4 is ‘1’. Otherwise, FPR>1.4 is ‘0’.
23. Create sold, died, removed, milk fever, retained placenta, metritis, ketosis, DA, pneumonia, diarrhea, lameness, foot trim, mastitis, and miscellaneous illness and other associated variables according to the definitions and thresholds in **S2 Appendix B**.
24. Days in milk are transformed to numeric objects. CLIVE, CSEX, CALF_SEX, FRESH_MONTH, FRESH 372 TO 7, FRESH 386 TO 21, FRESH 425 TO 60, ABORT, CALVING_EASE_>=2, CALVING_EASE_>=3, TWINS, STILLBIRTH, MALE_CALF, FPR>1.4, and all variable between SOLD and ILLMISC<=60 that does not end with ‘DIM’ are transformed to factor objects. The levels of RC, CLIVE, CSEX, CALF_SEX, FRESH_MONTH are sorted in alphabetical order. The levels of FRESH_MONTH, FRESH 372 TO 7, FRESH 386 TO 21, FRESH 425 TO 60, ABORT, CALVING_EASE_>=2, CALVING_EASE_>=3, TWINS, STILLBIRTH, MALE_CALF, FPR>1.4, and all variable between SOLD and ILLMISC<=60 that does not end with ‘DIM’ are defined such that ‘0’ is the reference level.
